# RCC1 Domain‐Containing Protein 1 Promotes Colon Cancer Malignant Progression by Activating Autophagy‐Dependent WNT5A Secretion in Cancer‐Associated Fibroblasts

**DOI:** 10.1002/snz2.70013

**Published:** 2026-01-18

**Authors:** Chao Liu, Sheng Xu, Yuanyuan Liu, Yuntian Tang

**Affiliations:** ^1^ Department of General Surgery The First Affiliated Hospital of Jinan University Guangzhou China; ^2^ Departments of Gastrointestinal, Hernia and Enterofistula Surgery The People's Hospital of Guangxi Zhuang Autonomous Region Nanning China; ^3^ Departments of Gynecology The People's Hospital of Guangxi Zhuang Autonomous Region Nanning China

**Keywords:** autophagy, cancer‐associated fibroblast, colon cancer, RCCD1, WNT5A

## Abstract

**Background:** Autophagy plays a dual role in colon cancer, but its mechanisms in tumor‐stroma interactions are unclear.

**Methods:** Using TCGA‐COAD and GSE161277 datasets, we identified autophagy‐related prognostic genes via bioinformatics and machine learning. RCCD1's role was further investigated using single‐cell RNA‐seq and functional coculture assays (fibroblasts/HCT116 cells), validated in clinical samples.

**Results:** RCCD1 was upregulated in colon cancer and cancer‐associated fibroblasts (CAFs), correlating with poor prognosis. In CAFs, RCCD1 activated AMPK/mTOR/ULK1 signaling, enhancing autophagy and driving WNT5A secretion. This activated the Wnt/CaMKII/ERK pathway in tumor cells, promoting EMT, proliferation, and invasion. These effects were reversed by autophagy inhibition or WNT5A neutralization.

**Conclusion:** The RCCD1‐autophagy‐WNT5A axis is a critical mediator of protumorigenic CAF‐tumor cell crosstalk, representing a novel therapeutic target for colon cancer.

## Graphical abstract

RCCD1 is upregulated in colon cancer and cancer‐associated fibroblasts (CAFs). In CAFs, RCCD1 overexpression activates AMPK/mTOR/ULK1 signaling, enhancing autophagy. This stimulates autophagic secretion of WNT5A. WNT5A then activates the nonclassical Wnt/CaMKII/ERK pathway in tumor cells, promoting epithelial‐mesenchymal transition (EMT), proliferation, invasion, and migration. Blockade of autophagy or WNT5A reverses these effects. The RCCD1‐autophagy‐WNT5A axis is a key mediator of protumorigenic CAF‐tumor crosstalk and a novel therapeutic target.

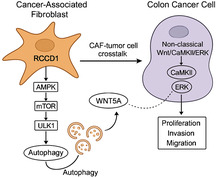

## 1 Introduction

Colon cancer represents a major digestive tract malignancy, ranking fifth globally in both incidence (6.0%) and mortality (5.8%) according to World Health Organization 2020 statistics (Yaghoubi et al. 2020). The disease shows an alarming trend of increasing incidence and younger onset, with patients under 50 years rising by 12% over the past decade (Karpisheh et al. 2019). Current treatment relies primarily on surgical resection supplemented by chemotherapy but faces significant challenges due to diagnostic difficulties—only 20% of patients present with localized disease (Dey et al. 2023), while most are diagnosed at advanced or metastatic stages (Fabregas et al. 2022). This late presentation limits curative surgical options and contributes to prominent chemotherapy resistance, resulting in poor treatment outcomes with 5‐year survival rates below 15% for patients with distant metastasis (Lahaye et al. 2024). These clinical realities highlight the urgent need to identify novel therapeutic targets and develop more effective treatment strategies to improve survival outcomes and quality of life for colon cancer patients.

Autophagy is a cellular self‐degradation process involving autophagolysosomes that maintains homeostasis by eliminating metabolic waste (Kocaturk et al. 2019). This mechanism is implicated in various human diseases including malignant tumors, liver damage, inflammatory conditions, and neurodegeneration (Antunes et al. 2018; Ortega et al. 2024). Accumulating evidence demonstrates that highly invasive tumors upregulate autophagy to promote survival (Nazio et al. 2019; Zamame Ramirez et al. 2021), making autophagy‐related genes crucial targets for cancer therapy (Zhang et al. 2023). In colon cancer, autophagy exhibits complex multilevel regulatory characteristics. Chaperone‐mediated autophagy (CMA) is abnormally activated, with significantly increased CMA markers (HSC70 and LAMP2A) in primary tumors potentially promoting progression through tumor suppressor protein degradation (Huang et al. 2023). Additionally, TP53 silencing in HCT116 colon cancer cells induces reticular and mitotic autophagy, maintaining mitochondrial homeostasis by clearing damaged organelles and enhancing chemotherapy resistance (Roshani‐Asl et al. 2020). Despite these insights, the precise mechanisms underlying autophagy's role in colon cancer development and progression remain incompletely understood.

This study aims to identify key autophagy‐related genes through comprehensive bioinformatics analysis and elucidate their functional roles in colon cancer progression, with particular focus on understanding how these genes regulate the crosstalk between cancer‐associated fibroblasts and tumor cells. We used differential expression analysis, weighted gene coexpression network analysis (WGCNA), and machine learning approaches to screen autophagy‐related prognostic genes from The Cancer Genome Atlas Colon Adenocarcinoma (TCGA) and Gene Expression Omnibus (GEO) databases, followed by single‐cell RNA sequencing analysis to determine cellular expression patterns. Clinical validation was performed using patient tissue samples, and mechanistic studies utilized coculture systems with cancer‐associated fibroblasts and colon cancer cells. Our findings identified RCC1 domain‐containing protein 1 (RCCD1) as a pivotal autophagy‐related gene that enhances autophagic flux in CAFs through the AMP‐activated protein kinase (AMPK)/mammalian target of rapamycin (mTOR)/unc‐51 like autophagy activating kinase 1 (ULK1) signaling axis, promoting autophagy‐dependent wingless‐type MMTV integration site family member 5A (WNT5A) secretion that subsequently activates the non‐classical Wnt/calmodulin‐dependent protein kinase II (CaMKII)/extracellular signal‐regulated kinase (ERK) pathway in tumor cells, thereby driving epithelial‐mesenchymal transition and malignant progression. This study reveals the core role of the RCCD1‐autophagy‐WNT5A axis in tumor‐stromal interactions, providing a novel therapeutic strategy for targeting the tumor microenvironment in colon cancer treatment.

## 2 Materials and Methods

### 2.1 Data Sources and Bioinformatics Analysis

RNA‐seq, clinical, and survival data were retrieved from The Cancer Genome Atlas (TCGA) database, including 473 colon adenocarcinoma (COAD) tissue samples with survival data and 41 normal tissue samples (TCGA‐COAD dataset), and the GSE161277 single‐cell RNA sequencing dataset was obtained from the Gene Expression Omnibus (GEO) database. Autophagy‐related genes were sourced from the GeneCards database. Differentially expressed genes (DEGs) between COAD patients and healthy controls were identified using the DESeq2 package (version 1.38.3) with threshold set at adjusted *p*‐value < 0.05 and |log2 fold change| > 1. Weighted gene coexpression network analysis (WGCNA) was performed using WGCNA package (version 1.70.3) to identify gene modules most correlated with COAD. For candidate feature selection, two machine learning methods were used: LASSO (Least Absolute Shrinkage and Selection Operator) regression was performed using the glmnet package with 10‐fold cross‐validation to identify optimal lambda parameters for feature selection, while random forest analysis was conducted using the randomForestSRC package with genes selected based on variable importance (vimp) > 0 using the var.select function. The intersection of DEGs, WGCNA‐identified genes, and autophagy‐related genes yielded potential key genes, which were further refined through the intersection of LASSO and random forest selected features to identify final candidate genes. These candidates were subjected to protein–protein interaction analysis using STRING database and functional enrichment analysis using clusterProfiler package (version 4.2.2). Single‐cell analysis was performed on GSE161277 dataset (25,516 cells) using Leiden clustering (resolution 0.6) and UMAP visualization, with cell–cell interaction analysis to evaluate fibroblast‐epithelial cell communication.

### 2.2 Clinical Sample Collection and Processing

Tumor tissues and adjacent normal tissues were collected from colon cancer patients at the Ethics Committee of The People's Hospital of Guangxi Zhuang Autonomous Region following institutional review board approval (IRB approval number: [lunli‐KY‐1IT‐2025‐72]) and written informed consent from all participants. Inclusion criteria were as follows: (1) histologically confirmed colon adenocarcinoma, (2) age ≥18 years, (3) no prior chemotherapy or radiotherapy, and (4) adequate tissue samples available for analysis. Exclusion criteria included the following: (1) patients with other malignancies, (2) severe comorbidities affecting treatment decisions, (3) incomplete clinical data, and (4) insufficient tissue quality for molecular analysis. Cancer‐associated fibroblasts (CAFs) were isolated from tumor tissues using Anti‐Fibroblast MicroBeads, human (Cat# 130‐050‐601, Miltenyi Biotec, Germany). Fresh tumor tissues were enzymatically dissociated into single‐cell suspensions using the Tumor Dissociation Kit, human (Cat# 130‐095‐929, Miltenyi Biotec) following manufacturer's protocol. The cell suspension was then passed through a 70 μm cell strainer (Cat# 352350, Corning) and incubated with Anti‐Fibroblast MicroBeads for 15 min at 4°C. Labeled cells were separated using an autoMACS Pro Separator (Miltenyi Biotec) with the “possel” separation program. Fibroblast purity was confirmed by flow cytometry analysis after the staining with a Human Fibroblast Activation Protein alpha/FAP PE‐conjugated Antibody (Cat#: FAB3715P, R&D Systems, USA) staining on BD FACSCanto II system (BD Biosciences, USA), achieving >95% purity after isolation.

### 2.3 Cell Culture and Transfection

HCT116 human colon cancer cells (Cat# CCL‐247, American Type Culture Collection, ATCC, USA) and CCD‐18Co normal human colon fibroblasts (Cat# CRL‐1459, ATCC) were cultured in DMEM medium (Cat# 11995065, Gibco, USA) supplemented with 10% fetal bovine serum (Cat# 16000044, FBS, Gibco) and 1% penicillin/streptomycin (Cat# 15140122, Gibco) at 37°C in 5% CO2. RCCD1 overexpression vector (OE‐RCCD1) was constructed in pcDNA3.1 backbone (Cat# V79020, Invitrogen, USA), and RCCD1‐specific siRNA (sh‐RCCD1) and control siRNA (sh‐NC) were purchased from GenePharma (Shanghai, China). Transfection was performed using Lipofectamine 3000 (Cat# L3000015, Invitrogen) according to manufacturer's instructions, with cells harvested 48–72 h post‐transfection for subsequent analyses.

### 2.4 Coculture Experiments and Drug Treatment

Transwell coculture system (0.4 μm pore size, Cat# 3460, Corning, USA) was established with CCD‐18Co fibroblasts (1 × 10^5 cells) in the upper chamber and HCT116 cells (1 × 10^5 cells) in the lower chamber. Experimental groups included HCT116 alone, coculture with sh‐NC, sh‐RCCD1, OE‐NC, OE‐RCCD1, OE‐RCCD1 + 3‐MA, and OE‐RCCD1 + anti‐WNT5A treatments. 3‐Methyladenine (3‐MA, Cat# M9281, Sigma–Aldrich, USA) was used at 5 mM concentration as autophagy inhibitor, and WNT5A neutralizing antibody (Cat# MAB645, R&D Systems, USA) was applied at 10 μg/mL concentration. Treatment duration was 48 h for all assays.

### 2.5 RNA Extraction and Real‐Time PCR

Total RNA was extracted using TRIzol reagent (Cat# 15596026, Invitrogen) and reverse transcribed using PrimeScript RT reagent kit (Cat# RR037A, Takara, Japan). Real‐time PCR was performed using SYBR Green Master Mix (Cat# RR820A, Takara) on ABI 7500 system (Cat# 4351105, Applied Biosystems), with GAPDH as internal control. Target genes included RCCD1, ATG5, BECN1, MAP1LC3B, ATG7, p62, E‐cadherin, Vimentin, and N‐cadherin. Relative expression levels were calculated using the 2^‐ΔΔCt method using GAPDH as an internal reference, and data are presented as fold changes compared to control groups.

### 2.6 Western Blot Analysis

Protein extraction was performed using Radio‐Immunoprecipitation Assay (RIPA) lysis buffer (Cat# P0013B, Beyotime Biotech, China) with protease inhibitors (Cat# 5892970001, Roche), and protein concentrations were determined by BCA assay (Cat# 23225, Pierce). Primary antibodies included anti‐RCCD1 (Cat# ab122570, Abcam, 1:1000), anti‐SQSTM1/p62 (Cat# 5114, Cell Signaling Technology (SCT), 1:1000), anti‐Beclin‐1 (Cat# 3495, CST, 1:1000), anti‐phospho‐mTOR (Ser2448) (Cat# 5536, CST, 1:1000), anti‐mTOR (Cat# 2983, CST, 1:1000), anti‐phospho‐AMPK*α* (Thr172) (Cat# 2535, CST, 1:1000), anti‐AMPK*α* (Cat# 5832, CST, 1:1000), anti‐phospho‐ULK1 (Ser757) (Cat# 14202, CST, 1:1000), anti‐ULK1 (Cat# 8054, CST, 1:1000), anti‐phospho‐CaMKII (Thr286) (Cat# 12716, CST, 1:1000), anti‐CaMKII (Cat# 4436, CST, 1:1000), anti‐phospho‐p44/42 MAPK (Erk1/2) (Cat# 4370, CST, 1:1000), anti‐p44/42 MAPK (Erk1/2) (Cat# 4695, CST, 1:1000), anti‐WNT5A (Cat# ab235966, Abcam, 1:1500), and anti‐*β*‐actin (Cat# 4970, CST, 1:2000). HRP‐conjugated secondary antibodies (anti‐rabbit IgG Cat# 7074, anti‐mouse IgG Cat# 7076, 1:5000, Cell Signaling Technology) were applied for 1 h at room temperature, and protein bands were visualized using enhanced chemiluminescence detection system (Cat# 32106, Pierce) with *β*‐actin as loading control.

### 2.7 Immunofluorescence Microscopy

Cells were fixed with 4% paraformaldehyde for 15 min, permeabilized with 0.1% Triton X‐100 for 10 min, and blocked with 5% BSA for 1 h at room temperature. Primary anti‐LC3B antibody (Cat# 3868, 1:500, Cell Signaling Technology) was applied overnight at 4°C, followed by Alexa Fluor 594‐conjugated secondary antibody (Cat# A‐11012, 1:400, Invitrogen) for 1 h at room temperature. Nuclei were counterstained with DAPI (Cat# D9542, Sigma–Aldrich), and images were captured using Olympus BX53 fluorescence microscope equipped with DP80 digital camera and cellSens imaging software (Olympus, Japan) with LC3B‐positive autophagosomes quantified in at least 100 cells per condition from multiple view fields of three independent experiments.

### 2.8 ELISA

WNT5A protein levels in culture supernatants and tissue lysates were measured using a Human WNT5A ELISA Kit (Colorimetric) (Cat# NBP3‐38894, Novus Biologicals, USA) according to manufacturer's instructions. Briefly, samples and standards were added to pre‐coated 96‐well plates and incubated for 2 h at room temperature, followed by detection antibody incubation and TMB substrate reaction (included in kit). Absorbance was measured at 450 nm with wavelength correction at 570 nm using a SpectraMax M5 microplate reader (Molecular Devices, USA) with SoftMax Pro software (version 7.1). WNT5A concentrations were calculated from standard curves using 4‐parameter logistic regression analysis, with results expressed as fold changes relative to the controls.

### 2.9 CCK‐8 Cell Growth Assay

Cell proliferation was assessed using Cell Counting Kit‐8 (Cat# CK04‐05, Dojindo, Japan) according to manufacturer's protocol. Cells were seeded at 3 × 10^3 per well in 96‐well plates and cultured under experimental conditions for indicated time periods. CCK‐8 solution (10 μL) was added to each well and incubated for 2 h at 37°C in 5% CO_2_. Absorbance was measured at 450 nm using a SpectraMax M5 microplate reader (Molecular Devices, USA).

### 2.10 Wound Healing Assay

Cells were seeded in 6‐well plates and cultured to 90%–95% confluence, then monolayers were scratched using sterile 200 μL pipette tips to create uniform wounds. Cells were washed twice with PBS to remove debris and cultured in serum‐free medium to minimize proliferation effects. Wound closure was monitored and photographed at 0 and 48 h using phase‐contrast microscopy, with migration rate calculated as percentage of wound area closure compared to initial wound width.

### 2.11 Transwell Invasion Assay

Cell invasion was evaluated using Matrigel‐coated Transwell chambers (8 μm pore size, Cat# 3422, Corning) according to standard protocol. Briefly, 5 × 10^4 cells in 200 μL serum‐free medium were seeded into the upper chambers pre‐coated with Matrigel (Cat# 354234, BD Biosciences), while 600 μL complete medium containing 10% FBS was added to lower chambers as chemoattractant. After 24‐h incubation at 37°C, noninvaded cells on the upper surface were removed with cotton swabs, and invaded cells were fixed with 4% paraformaldehyde, stained with 0.1% crystal violet (Cat# C0775, Sigma–Aldrich), and counted under light microscopy in five random fields per chamber.

### 2.12 Statistical Analysis

Data are presented as mean ± standard deviation from at least three independent experiments. Data analyses were performed using the GraphPad Prism 8.0 software with Student's t‐test or Wilcoxon rank‐sum test for two‐group comparisons. One‐way ANOVA followed by Tukey's post‐hoc test was applied for multiple group comparisons. Two‐way ANOVA with Tukey's post‐hoc test was used for time‐course proliferation assays with multiple groups and time points. Survival analysis was conducted using log‐rank test with Kaplan–Meier curves generated using survival package in R. *p*‐values <0.05 were considered statistically significant (*p < 0.05, **p < 0.01, ***p < 0.001, ****p < 0.0001).

## 3 Results

### 3.1 Identification of Autophagy‐Related Candidate Genes Through Integrated Bioinformatics Analysis

**FIGURE 1:**
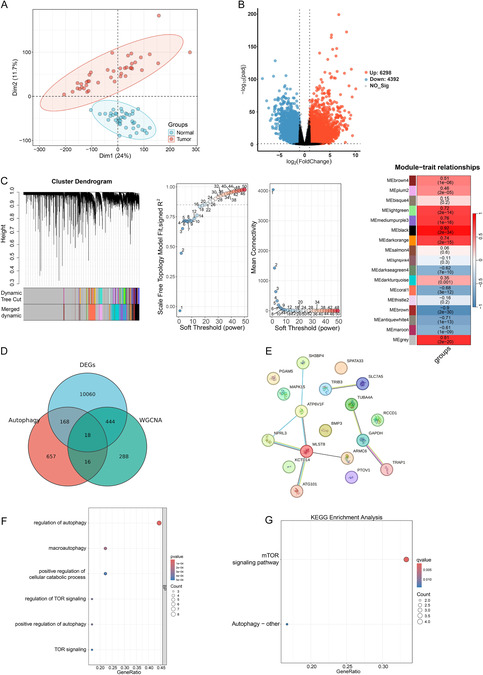
Identification of autophagy‐related candidate genes through integrated bioinformatics analysis. TCGA‐COAD dataset (*n* = 41 normal, *n* = 473 tumor samples) was analyzed using integrated bioinformatics approaches. (A) Principal component analysis (PCA) of sample distribution between normal and tumor groups. (B) Volcano plot of differential gene expression analysis performed using DESeq2 package (version 1.38.3) with thresholds: adjusted *p* < 0.05 and |log2FC| > 1. Red dots: upregulated genes; blue dots: downregulated genes; gray dots: nonsignificant genes. (C) Weighted gene coexpression network analysis (WGCNA) showing gene module identification and module‐trait relationships. Left panel: Gene clustering dendrogram based on topological overlap measure (TOM) dissimilarity. The colored bars below represent different gene modules identified by dynamic tree cutting algorithm, with each color corresponding to a distinct coexpression module. Middle panel: Scale independence (upper) and mean connectivity (lower) plots for soft‐threshold power selection. The plots help determine the optimal soft‐thresholding power (*β*) for constructing the network. The *x*‐axis shows soft‐threshold values, while *y*‐axis shows the scale‐free topology fit index (*R* ^2^) and mean connectivity respectively. Right panel: Module‐trait relationship heatmap showing correlations between identified gene modules (rows) and clinical traits (columns). The color intensity corresponds to the correlation coefficient (ranging from −1 to 1), with red indicating positive correlation and blue indicating negative correlation. Each cell contains the correlation coefficient and its corresponding *p*‐value in parentheses. The analysis was performed using WGCNA package (version 1.70.3) in *R*. (D) Venn diagram showing three‐way intersection of differentially expressed genes, WGCNA black module genes, and autophagy‐related genes from GeneCards database. (E) Protein–protein interaction network of 18 candidate genes constructed using STRING database (confidence score > 0.4). Node size indicates connection degree; edge thickness represents interaction confidence. (F) Gene Ontology enrichment analysis of candidate genes performed using clusterProfiler package (version 4.2.2). Dot size indicates gene count; color represents adjusted *p*‐value. (G) KEGG pathway enrichment analysis. Dot size indicates gene count; color represents adjusted *p*‐value. Statistical analysis: DESeq2 for differential expression; Pearson correlation for WGCNA; hypergeometric test with Benjamini–Hochberg correction for enrichment analyses.

We initially aimed to systematically identify autophagy‐related genes with potential prognostic significance in colon adenocarcinoma through comprehensive bioinformatics screening. Principal component analysis (PCA) demonstrated clear sample separation between normal and COAD groups, confirming the reliability of the TCGA dataset for subsequent differential expression analysis (Figure 1A). To characterize the molecular landscape of colon cancer, differential expression analysis of TCGA‐COAD samples identified 10,690 differentially expressed genes (DEGs), comprising 6298 upregulated genes (red) and 4392 downregulated genes (blue) (Figure 1B, volcano plot). Subsequently, weighted gene coexpression network analysis (WGCNA) was used to identify disease‐relevant gene modules, revealing that the black module containing 766 genes exhibited the strongest correlation with COAD phenotype (Figure 1C). To specifically focus on autophagy‐related mechanisms in colon cancer progression, we performed a three‐way intersection of DEGs (Normal vs COAD), WGCNA black module genes, and autophagy‐related genes from the GeneCards database, which yielded 18 potential key candidate genes (Figure 1D). Further characterization through protein–protein interaction network analysis using STRING database revealed complex interconnections among these candidate genes, suggesting coordinated regulatory mechanisms (Figure 1E). Finally, Gene Ontology (GO) and Kyoto Encyclopedia of Genes and Genomes (KEGG) enrichment analyses confirmed that the identified candidate genes were significantly enriched in autophagy‐related biological processes and signaling pathways, thereby validating our integrated screening strategy and supporting their potential roles in autophagy‐mediated colon cancer pathogenesis (Figure 1F,G).

### 3.2 Machine Learning‐Based Feature Selection Identifies RCCD1 as the Key Autophagy‐Related Gene

**FIGURE 2:**
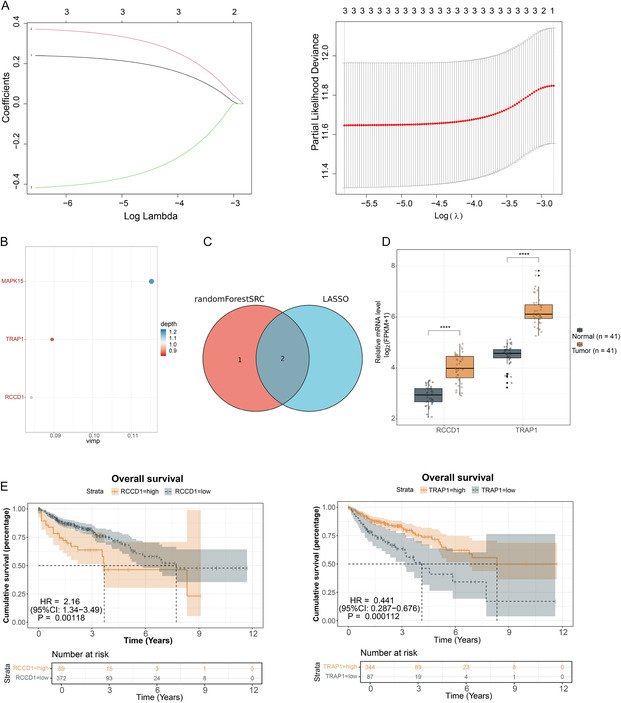
Machine learning‐based feature selection identifies RCCD1 as the key autophagy‐related gene. The 18 candidate genes were subjected to machine learning analysis using TCGA‐COAD dataset. (A) LASSO regression analysis performed using glmnet package with 10‐fold cross‐validation. Left: coefficient profiles against log(*λ*); Right: cross–validation curve showing MSE ± SD. Vertical dashed lines indicate optimal *λ* value. (B) Random forest variable importance analysis conducted using randomForestSRC package. Genes with vim *p* > 0 were selected using var.select function. (C) Venn diagram of genes selected by LASSO and random forest methods. (D) Box plots comparing RCCD1 and TRAP1 expression levels between paired normal and tumor tissues from the TCGA‐COAD cohort (*n* = 41 matched pairs). Box boundaries: 25th–75th percentiles; center line: median; whiskers: 1.5× interquartile range. (E) Kaplan–Meier survival curves stratifying patients by median expression of RCCD1 and TRAP1. Shaded areas: 95% confidence intervals; numbers below: patients at risk. Statistical analysis: Wilcoxon rank‐sum test for expression comparisons (****p < 0.0001); log‐rank test for survival analysis (*p* < 0.05).

To further refine the candidate gene selection, we used two complementary machine learning approaches for feature selection. LASSO regression analysis with 10‐fold cross‐validation identified an optimal lambda value and selected two genes with nonzero coefficients (Figure 2A), while random forest analysis based on variable importance scores isolated three genes with vim *p* > 0 (Figure 2B). The intersection of genes selected by both machine learning methods yielded two final candidate genes: RCCD1 and TRAP1 (Figure 2C). Expression analysis demonstrated that both candidate genes were significantly upregulated in tumor tissues compared to normal tissues (Figure 2D). Kaplan–Meier survival analysis revealed distinct prognostic impacts for each gene: patients with high RCCD1 expression exhibited significantly shorter overall survival (*p* < 0.05), whereas patients with low TRAP1 expression paradoxically showed poorer prognosis (*p* < 0.05) (Figure 2E). Given that RCCD1 demonstrated both consistent upregulation in tumors and clear association with poor prognosis in high‐expression patients, RCCD1 was selected as the final key gene for subsequent mechanistic investigations.

### 3.3 Single‐Cell Analysis Identifies RCCD1 Overexpression in Cancer‐Associated Fibroblasts

**FIGURE 3:**
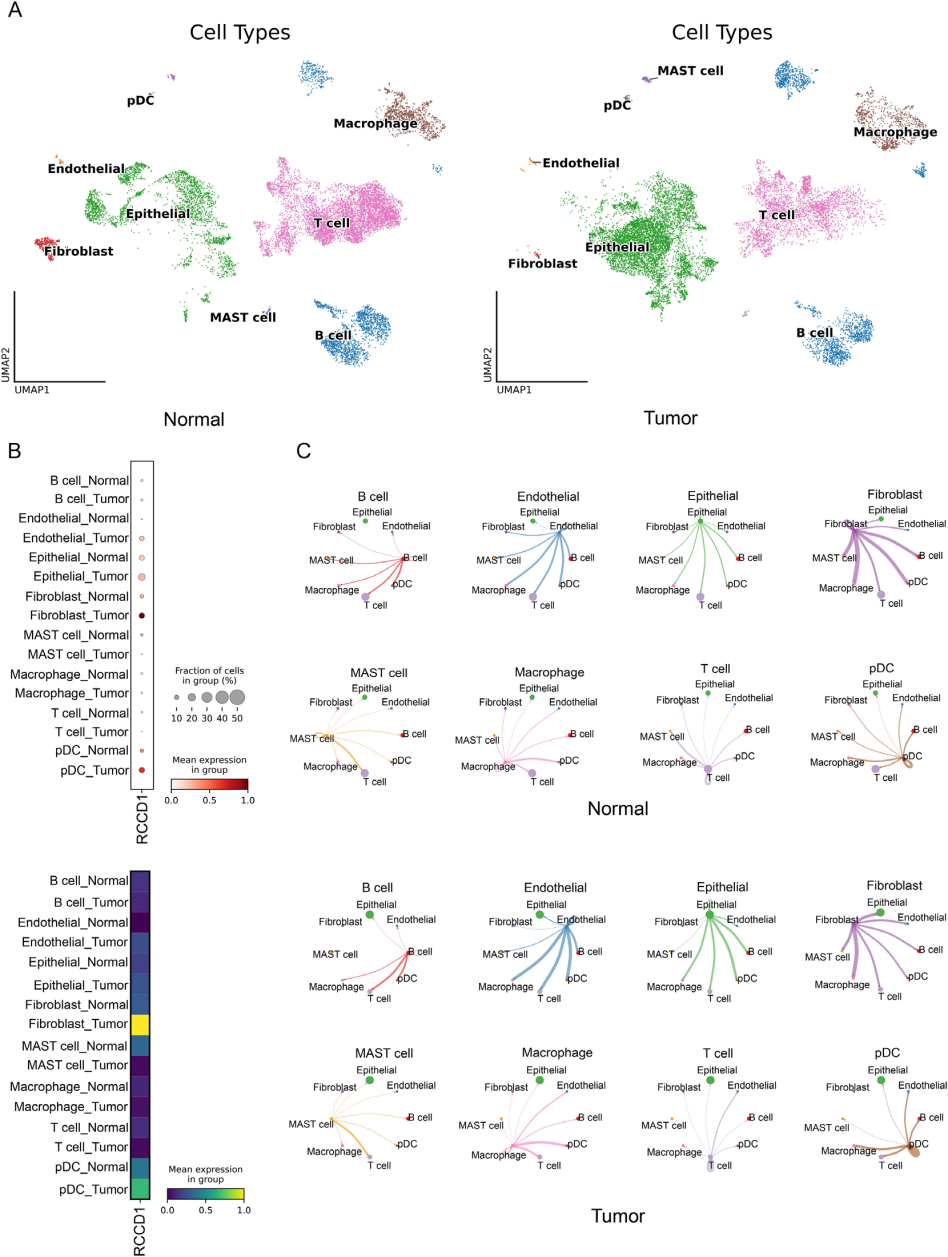
Single‐cell analysis identifies RCCD1 overexpression in cancer‐associated fibroblasts. GSE161277 single‐cell RNA sequencing dataset (25,516 cells) was analyzed. (A) UMAP plot showing eight cell populations identified by Leiden clustering (resolution 0.6). Cell types annotated based on canonical marker gene expression. (B) Feature plot showing RCCD1 expression levels across cell types. Color intensity represents normalized log‐transformed expression. (C) Cell–cell interaction analysis between fibroblasts and epithelial cells and other cell types in normal versus tumor tissues. Line thickness represents interaction strength calculated from ligand‐receptor pair expression levels. Statistical analysis: Wilcoxon rank‐sum test for differential expression comparisons; permutation tests for cell–cell interaction significance (analysis performed using CellChat or CellPhoneDB tools).

Single‐cell RNA sequencing analysis of GSE161277 dataset yielded 25,516 high‐quality cells after quality control. Leiden clustering (resolution 0.6) identified eight distinct cell populations visualized by UMAP (Figure 3A). RCCD1 expression analysis revealed predominant upregulation in cancer‐associated fibroblasts (CAFs) compared to other cell types (Figure 3B). Cell–cell interaction analysis demonstrated enhanced communication between fibroblasts and epithelial cells in tumor tissues (Figure 3C), suggesting that RCCD1 overexpression in CAFs may promote tumor progression through intercellular crosstalk.

### 3.4 Clinical Validation Confirms RCCD1 Upregulation and Enhanced Autophagy in Tumor‐Associated Fibroblasts

**FIGURE 4:**
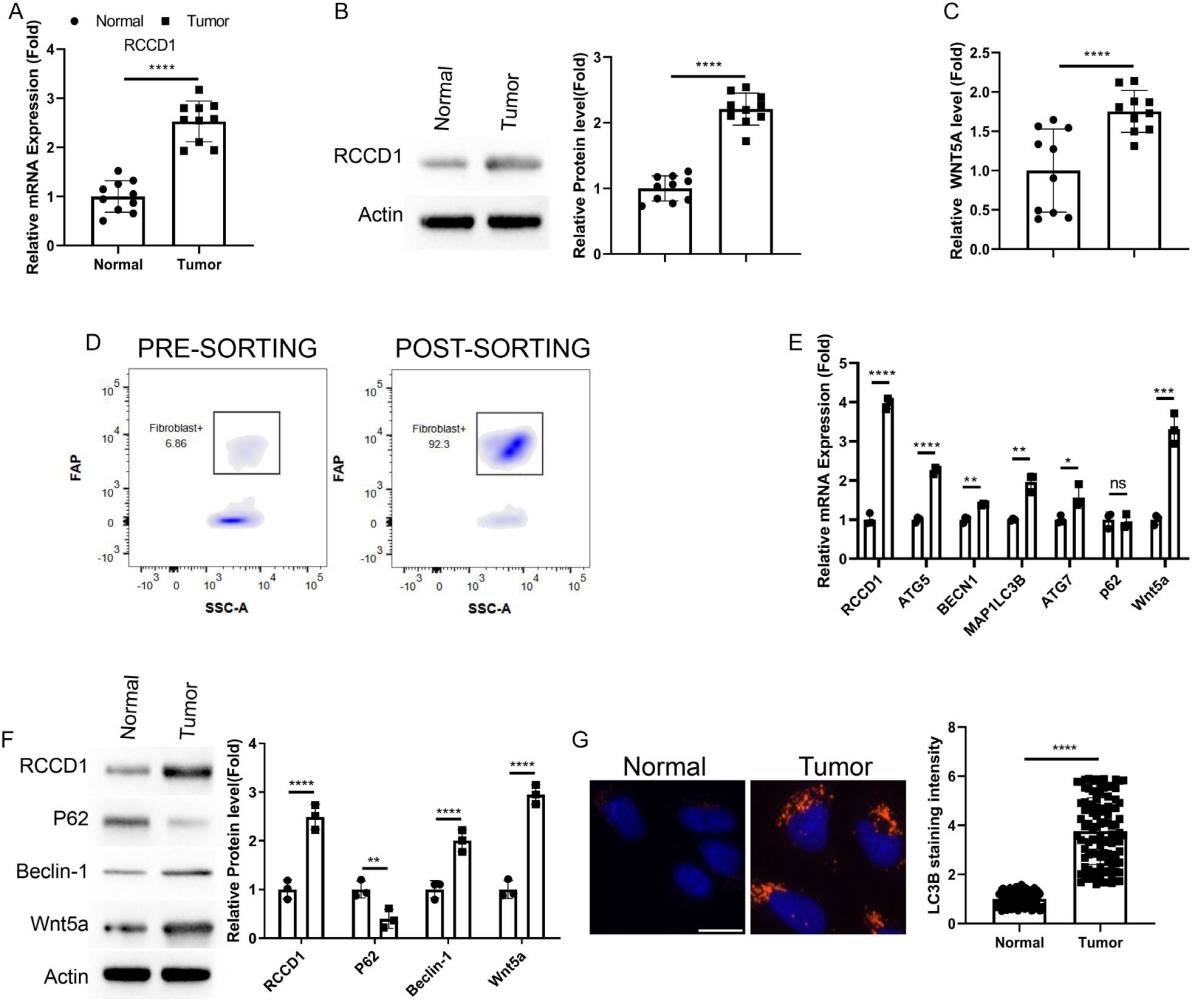
Clinical validation confirms RCCD1 upregulation and enhanced autophagy in tumor‐associated fibroblasts. Paired tumor and adjacent normal tissues from colon cancer patients were analyzed, with fibroblasts isolated using magnetic bead separation. (A) RT‐qPCR analysis of RCCD1 mRNA expression in tissue lysates. Expression normalized to GAPDH using 2^‐ΔΔCt method. (B) Western blot analysis of RCCD1 protein in tissue lysates with *β*‐actin loading control. Quantification by densitometry normalized to *β*‐actin. (C) ELISA quantification of WNT5A protein in tissue lysates using Human WNT5A ELISA Kit. (D) Flow cytometry validation of fibroblast purity using FAP staining on BD FACSCanto II system. Gates show FAP + cell percentages. (E) RT‐qPCR analysis of autophagy genes (ATG5, BECN1, MAP1LC3B, ATG7, and p62), RCCD1, and WNT5A in isolated fibroblasts. Expression normalized to GAPDH using 2^‐ΔΔCt method. (F) Western blot analysis of RCCD1, WNT5A, Beclin‐1, and p62 in isolated fibroblasts. Quantification by densitometry normalized to *β*‐actin. (G) LC3B immunofluorescence in isolated fibroblasts. Nuclear stain: DAPI (blue). Quantification from ≥100 cells per condition. Scale bar: 15 µm. Statistical analysis: t‐test for all comparisons; data presented as mean ± SD from *n* = 10 patient samples in A–C; data presented as mean ± SD from 3 experiments using 3 batchs of isolsated fibroblasts in D‐G. *p < 0.05; **p < 0.01, ***p < 0.001, ****p < 0.0001.

To validate our bioinformatics findings, we collected paired tumor and adjacent normal tissues from colon cancer patients for comprehensive molecular analysis. RT‐qPCR analysis confirmed significant upregulation of RCCD1 mRNA expression in tumor tissues compared to normal tissues (Figure 4A), which was further corroborated at the protein level (Figure 4B). Given the established role of WNT5A in colon cancer development (Sun et al. 2021), ELISA analysis was performed and revealed substantially elevated WNT5A protein levels in tumor tissues relative to normal controls (Figure 4C).

To specifically examine RCCD1 expression and autophagy activity in stromal fibroblasts, we isolated fibroblasts from both tumor and normal tissues using magnetic bead separation, achieving high purity as confirmed by flow cytometry analysis (Figure 4D). Molecular characterization of isolated fibroblasts revealed elevated RCCD1 expression in tumor‐associated fibroblasts compared to normal fibroblasts, accompanied by significant upregulation of key autophagy genes (ATG5, BECN1, MAP1LC3B, and ATG7) and WNT5A, while p62 levels remained unchanged (Figure 4E). Protein‐level analysis demonstrated increased RCCD1, WNT5A and Beclin‐1 expression alongside decreased p62 levels in CAFs, consistent with enhanced autophagic flux (Figure 4F). Immunofluorescence staining for LC3B further confirmed increased autophagosome formation in CAFs, as evidenced by enhanced formation of LC3B‐positive autophagic vesicles (Figure 4G). These results collectively validate RCCD1 overexpression in CAFs and establish its association with enhanced autophagy activity specifically in CAFs.

### 3.5 RCCD1 Modulates Autophagy Activity and WNT5A Secretion in Fibroblasts

**FIGURE 5:**
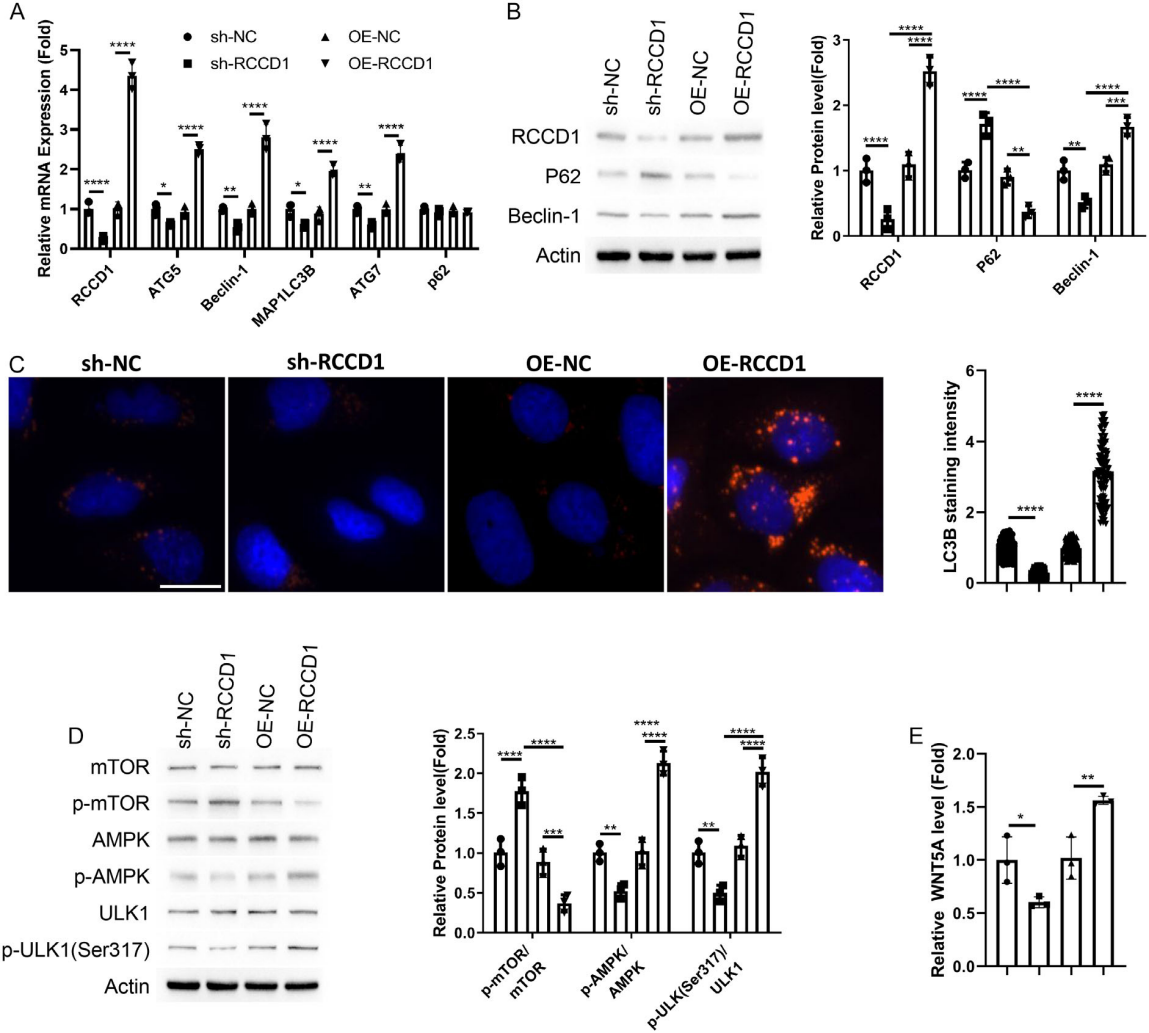
RCCD1 modulates autophagy activity and WNT5A secretion in CCD‐18Co fibroblasts. CCD‐18Co fibroblasts were transfected with sh‐RCCD1 (shRNA targeting RCCD1), sh‐NC (control shRNA), OE‐RCCD1 (RCCD1 expression vector), or OE‐NC (empty vector) constructs. Cells harvested 48 h post‐transfection. (A) RT‐qPCR analysis of autophagy genes (ATG5, BECN1, MAP1LC3B, ATG7, and p62). Expression normalized to GAPDH using 2^‐ΔΔCt method. (B) Western blot analysis of RCCD1, Beclin‐1, and p62 with *β*‐actin loading control. (C) LC3B immunofluorescence in isolated fibroblasts. Nuclear stain: DAPI (blue). Quantification from ≥100 cells per condition. Scale bar: 15 µm. (D) Western blot analysis of phosphorylated and total AMPK (Thr172), mTOR (Ser2448), and ULK1 (Ser555). Quantification shows phospho‐to‐total protein ratios. (E) ELISA quantification of WNT5A in culture supernatants using Human WNT5A ELISA Kit. Statistical analysis: one‐way ANOVA with Tukey's post‐hoc test; data presented as mean ± SD from *n* = 3 independent experiments. *p < 0.05, **p < 0.01, ***p < 0.001, ****p < 0.0001.

We next investigated RCCD1's functional role by establishing knockdown and overexpression models in CCD‐18Co fibroblasts. RT‐qPCR analysis revealed that RCCD1 knockdown significantly reduced autophagy genes (ATG5, BECN1, MAP1LC3B, and ATG7), while overexpression enhanced their expression, with p62 levels unchanged (Figure 5A). Western blot analysis confirmed these findings, showing that RCCD1 knockdown decreased RCCD1 and Beclin‐1 while increasing p62 accumulation, whereas overexpression showed opposite effects (Figure 5B). LC3B immunofluorescence demonstrated that RCCD1 knockdown reduced autophagosome formation while overexpression enhanced this process (Figure 5C). Mechanistically, RCCD1 knockdown increased mTOR phosphorylation and decreased AMPK/ULK1 phosphorylation, while overexpression showed inverse effects, indicating RCCD1 promotes autophagy through AMPK/mTOR/ULK1 signaling (Figure 5D). Additionally, ELISA analysis revealed that RCCD1 expression positively correlated with WNT5A secretion (Figure 5E), establishing RCCD1 as a critical regulator of both autophagy activity and WNT5A production in fibroblasts.

### 3.6 RCCD1 Overexpression in CCD‐18Co Fibroblasts Promotes Colon Cancer Cell Malignant Behaviors Through Epithelial‐Mesenchymal Transition (EMT)

**FIGURE 6:**
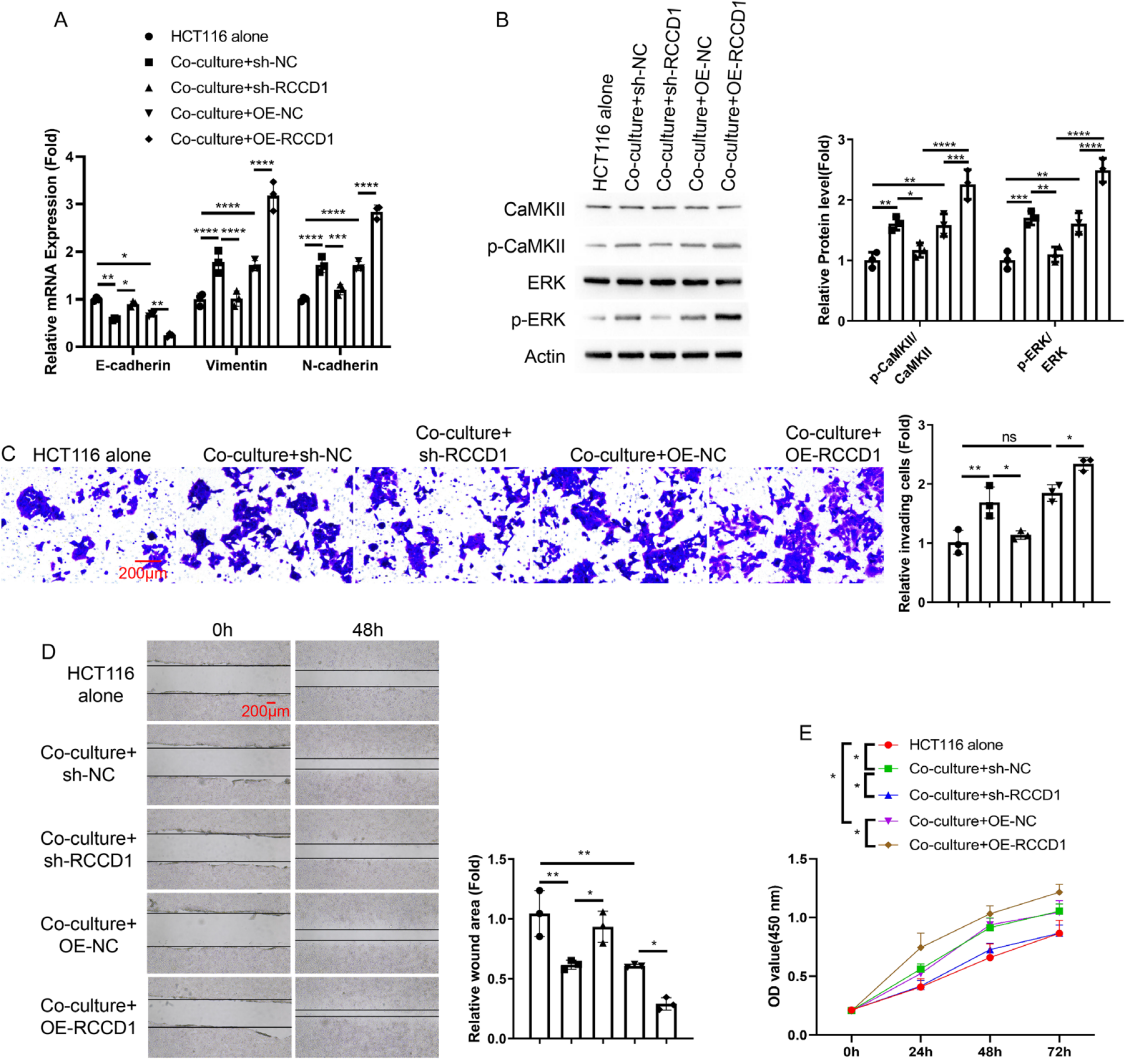
RCCD1 overexpression in CCD‐18Co fibroblasts promotes colon cancer cell malignant behaviors through epithelial‐mesenchymal transition induction. Transwell coculture system (0.4 μm pore, Corning): CCD‐18Co fibroblasts (upper chamber) transfected with sh‐NC, sh‐RCCD1, OE‐NC, or OE‐RCCD1; HCT116 cells (lower chamber). Five groups: HCT116 alone, coculture with sh‐NC, sh‐RCCD1, OE‐NC, or OE‐RCCD1 fibroblasts. Coculture duration: 48 h. All assays performed on HCT116 cells. (A) RT‐qPCR analysis of EMT markers (E‐cadherin, Vimentin, and N‐cadherin) in HCT116 cells. Expression normalized to GAPDH using 2^‐ΔΔCt method. (B) Western blot analysis of phosphorylated and total CaMKII (Thr286) and ERK1/2 (Thr202/Tyr204) with *β*‐actin loading control. Quantification shows phospho‐to‐total ratios. (C) Transwell invasion assay using Matrigel‐coated chambers: HCT116 cells were seeded in serum‐free medium; lower chamber contained 10% FBS. After 24 h, invaded cells were stained and counted in five random fields per condition. (D) Wound healing assay: confluent monolayers scratched with 200 μL pipette tips and cultured in serum‐free medium. Images captured at 0 and 48 h. Quantification shows relative wound areas. (E) CCK‐8 proliferation assay in different groups of HCT116 cells at 0, 24, 48, and 72 h. Absorbance was measured at 450 nm. Statistical analysis: one‐way ANOVA with Tukey's post‐hoc test for panels A–D; two‐way ANOVA with Tukey's post‐hoc test for panel E; data presented as mean ± SD from *n* = 3 independent experiments. *p < 0.05, **p < 0.01, ***p < 0.001, ****p < 0.0001.

To investigate whether RCCD1‐expressing fibroblasts influence colon cancer cell behavior, we performed coculture experiments using HCT116 cells with RCCD1‐modified CCD‐18Co fibroblasts. EMT marker analysis revealed that coculture with CCD‐18Co fibroblasts induced mesenchymal transition in HCT116 cells, characterized by decreased E‐cadherin and increased Vimentin and N‐cadherin expression. RCCD1 knockdown in CCD‐18Co fibroblasts partially reversed this EMT phenotype, while RCCD1 overexpression further enhanced mesenchymal marker expression (Figure 6A). Mechanistically, coculture activated CaMKII and ERK signaling in HCT116 cells, with RCCD1 knockdown reducing and overexpression enhancing these phosphorylation events (Figure 6B). Functionally, coculture with CCD‐18Co fibroblasts significantly enhanced HCT116 cell proliferation, invasion, and migration abilities as measured by CCK‐8, Transwell, and wound healing assays, respectively. RCCD1 knockdown in CCD‐18Co fibroblasts attenuated these malignant behaviors while overexpression further promoted them (Figure 6C–E). These results establish that RCCD1 in CCD‐18Co fibroblasts promotes colon cancer cell malignant behaviors through EMT induction and activation of CaMKII/ERK signaling pathways.

### 3.7 Autophagy Inhibition Attenuates RCCD1‐Induced Autophagy in Fibroblasts

**FIGURE 7:**
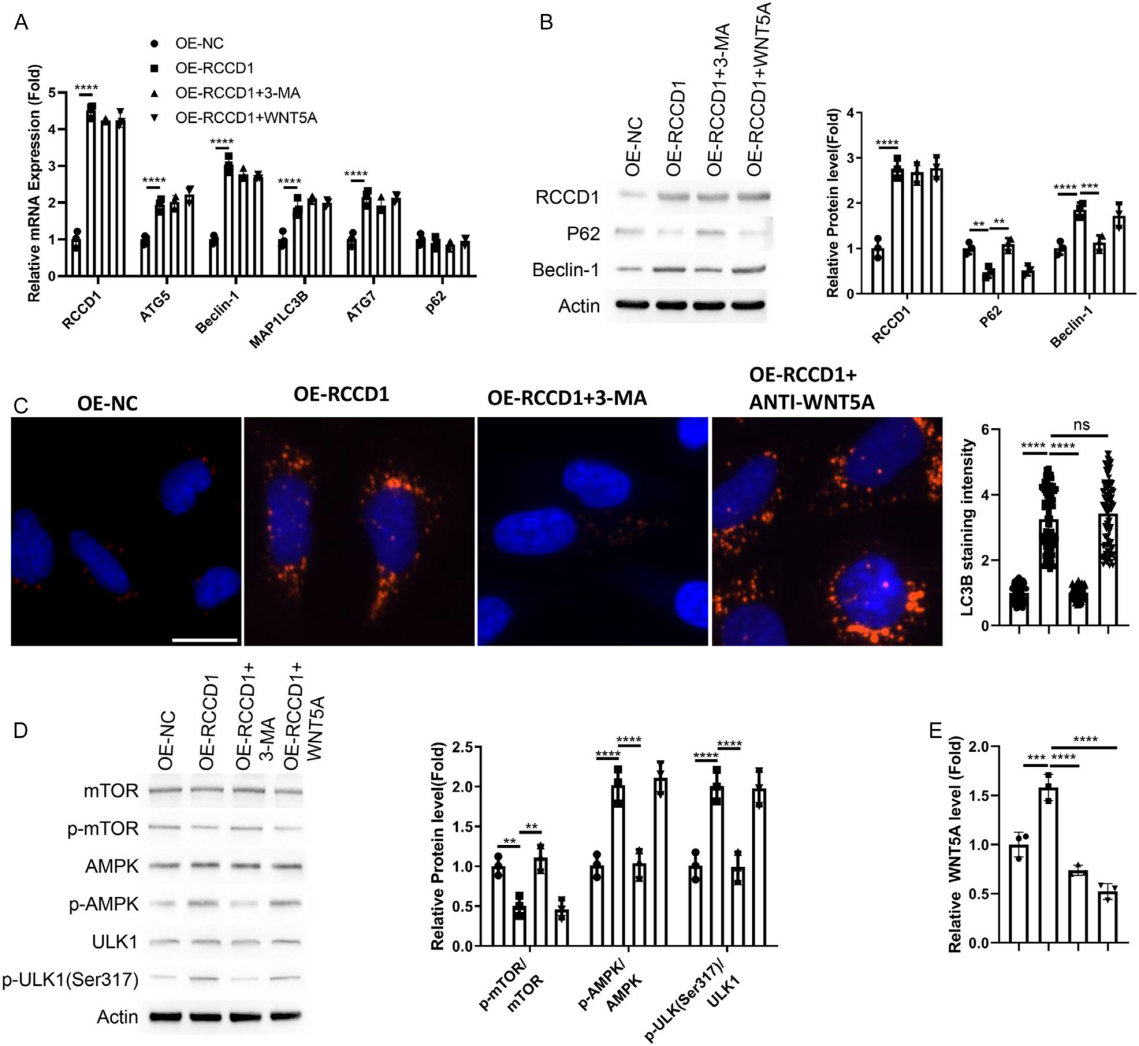
Autophagy inhibition attenuates RCCD1‐induced autophagy in CCD‐18Co fibroblasts. CCD‐18Co fibroblasts transfected with OE‐NC or OE‐RCCD1 were treated with vehicle, 3‐MA (autophagy inhibitor, 5 mM), or anti‐WNT5A antibody (10 μg/mL) for 48 h. (A) RT‐qPCR analysis of autophagy genes (ATG5, BECN1, MAP1LC3B, ATG7, and p62). Expression normalized to GAPDH using 2^‐ΔΔCt method. (B) Western blot analysis of RCCD1, Beclin‐1, and p62 with *β*‐actin loading control. Quantification by densitometry. (C) LC3B immunofluorescence in isolated fibroblasts. Nuclear stain: DAPI (blue). Quantification from ≥100 cells per condition. Scale bar: 15 µm. (D) Western blot analysis of phosphorylated and total AMPK (Thr172), mTOR (Ser2448), and ULK1 (Ser555). Quantification shows phospho‐to‐total ratios. (E) ELISA quantification of WNT5A in culture supernatants. Statistical analysis: one‐way ANOVA with Tukey's post‐hoc test; data presented as mean ± SD from *n* = 3 independent experiments. **p < 0.01, ***p < 0.001, ****p < 0.0001.

To elucidate RCCD1's mechanistic pathway, we treated RCCD1‐overexpressing CCD‐18Co fibroblasts with 3‐MA (autophagy inhibitor) and anti‐WNT5A antibody. RCCD1 overexpression upregulated autophagy genes (Figure 7A) and proteins while enhancing autophagosome formation (Figure 7B,C). Mechanistically, RCCD1 decreased mTOR phosphorylation while increasing AMPK/ULK1 phosphorylation (Figure 7D) and enhanced WNT5A secretion (Figure 7E). 3‐MA treatment reversed RCCD1‐induced autophagy effects and reduced WNT5A secretion, while anti‐WNT5A neutralization showed no effects on fibroblasts, indicating that WNT5A functions as a paracrine factor. These findings indicate that RCCD1 promotes autophagy possibly through AMPK/mTOR/ULK1 signaling in fibroblasts, and WNT5A secretion is regulated by RCCD1‐mediated autophagy.

### 3.8 Autophagy Inhibition and WNT5A Neutralization Reverse RCCD1‐Induced Fibroblast‐Mediated Cancer Cell Malignant Behaviors

**FIGURE 8:**
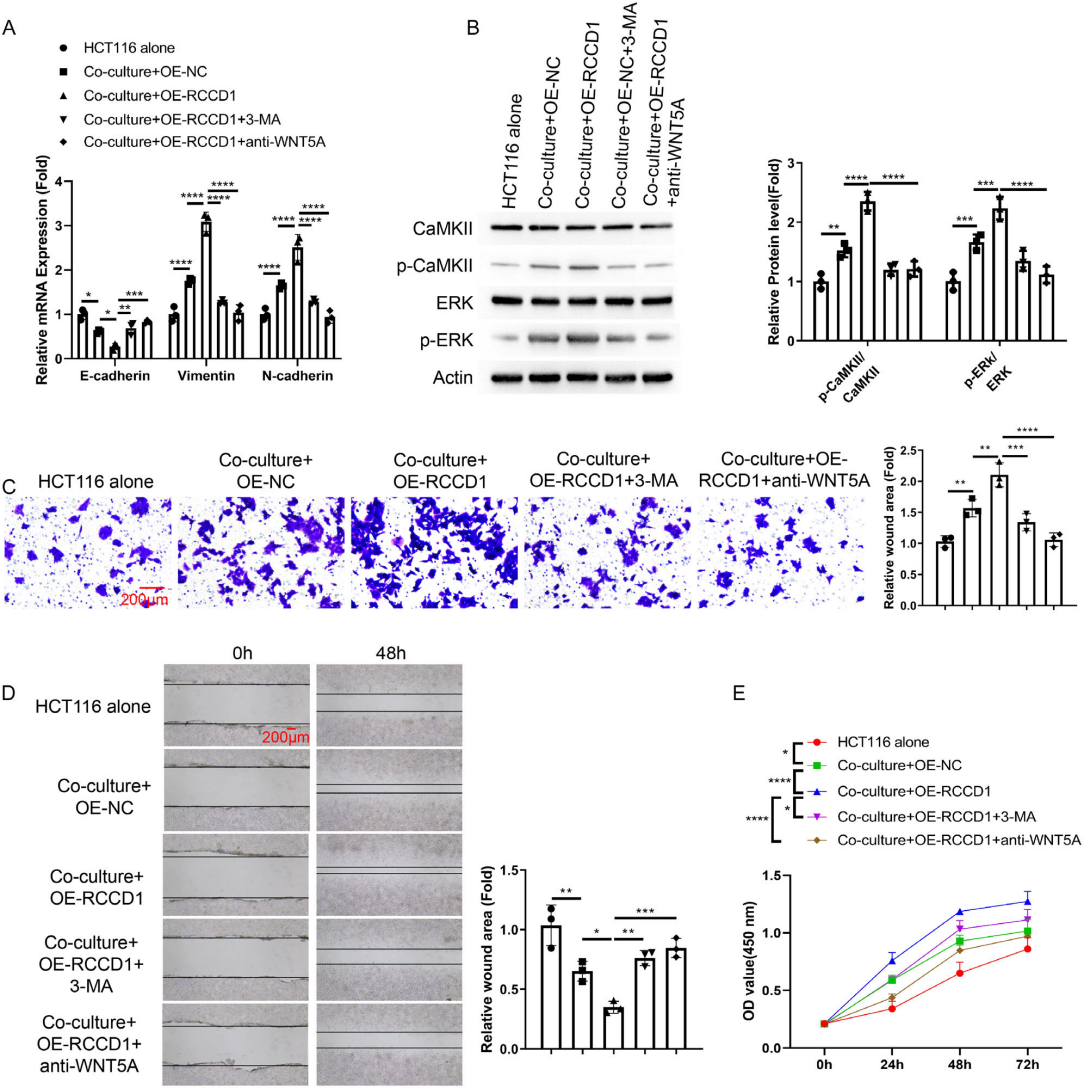
Autophagy inhibition and WNT5A neutralization reverse RCCD1‐induced fibroblast‐mediated cancer cell malignant behaviors. Transwell coculture system: CCD‐18Co fibroblasts (upper chamber) transfected with OE‐NC or OE‐RCCD1, treated with vehicle, 3‐MA (5 mM), or anti‐WNT5A antibody (10 μg/mL); HCT116 cells (lower chamber). Five groups: HCT116 alone, coculture with OE‐NC, OE‐RCCD1, OE‐RCCD1 + 3‐MA, or OE‐RCCD1 + anti‐WNT5A fibroblasts. Coculture duration: 48 h. All assays performed on HCT116 cells. (A) RT‐qPCR analysis of EMT markers (E‐cadherin, Vimentin, and N‐cadherin). Expression normalized to GAPDH using 2^‐ΔΔCt method. (B) Western blot analysis of phosphorylated and total CaMKII (Thr286) and ERK1/2 (Thr202/Tyr204) with *β*‐actin loading control. Quantification shows phospho‐to‐total ratios. (C) Transwell invasion assay in Matrigel‐coated chambers using different groups of HCT116 cells for 24 h. Invaded cells were stained with crystal violet and counted in five random fields. (D) Wound healing assay in scratched monolayers cultured in serum‐free medium; images at 0 and 48 h. Quantification shows relative wound areas. (E) CCK‐8 proliferation assay in different groups of HCT116 cells at 0, 24, 48, and 72 h. Absorbance was measured at 450 nm. Statistical analysis: one‐way ANOVA with Tukey's post‐hoc test for panels A–D; two‐way ANOVA with Tukey's post‐hoc test for panel E; data presented as mean ± SD from *n* = 3 independent experiments. *p < 0.05, **p < 0.01, ***p < 0.001, ****p < 0.0001.

To validate the role of RCCD1‐mediated autophagy and WNT5A signaling in cancer progression, we performed rescue experiments using cocultures of HCT116 cells with RCCD1‐overexpressing CCD‐18Co fibroblasts treated with 3‐MA or anti‐WNT5A. EMT marker analysis revealed that coculture with control fibroblasts decreased E‐cadherin and increased Vimentin and N‐cadherin in HCT116 cells, with RCCD1 overexpression further enhancing this mesenchymal transition. Both 3‐MA and anti‐WNT5A treatments significantly reversed the EMT phenotype by restoring E‐cadherin and reducing mesenchymal markers (Figure 8A). Mechanistically, CaMKII and ERK phosphorylation were progressively increased by coculture and RCCD1 overexpression but were effectively attenuated by both 3‐MA and anti‐WNT5A treatments (Figure 8B). Functionally, HCT116 cell proliferation, invasion, and migration abilities were sequentially enhanced by coculture with control fibroblasts and further promoted by RCCD1 overexpression. However, both autophagy inhibition with 3‐MA and WNT5A neutralization with anti‐WNT5A significantly reduced these malignant behaviors (Figure 8C–E). These results demonstrate that RCCD1‐mediated autophagy in fibroblasts promotes cancer cell malignant behaviors through WNT5A‐dependent paracrine signaling.

## 4 Discussion

Colon cancer remains the second leading cause of cancer‐related deaths globally, with approximately 500,000 annual deaths and 5‐year survival rates below 15% for advanced cases (Karpisheh et al. 2019; Yaghoubi et al. 2020). Despite advances in targeted therapies including anti‐EGFR and anti‐VEGF agents, and immunotherapy for dMMR/MSI‐H subtypes (comprising ∼15% of all colon cancer cases but only ∼5% of metastatic cases eligible for PD‐1 based therapy), treatment efficacy remains limited due to tumor heterogeneity and drug resistance (Dienstmann et al. 2015; Loria et al. 2024; Tomášek and Fiala 2025). These challenges underscore the urgent need for novel therapeutic strategies targeting tumor microenvironment mechanisms. Autophagy, a lysosomal degradation pathway maintaining cellular homeostasis, exhibits dual roles in cancer by initially suppressing tumorigenesis but subsequently promoting tumor survival, metastasis, and treatment resistance (Devenport and Shah 2019; Hu et al. 2016; Roshani‐Asl et al. 2020; Tanabe et al. 2011; Taraborrelli et al. 2023; Xiao et al. 2018; Yu et al 2022; Zhu et al. 2020). Recent studies have also found that autophagy is closely related to the tumor microenvironment (TME), immune escape, and treatment resistance and has become an important target in cancer research (Hu et al. 2016; Taraborrelli et al. 2023; Zhu et al. 2020). Our identification of the RCCD1‐autophagy‐WNT5A axis in cancer‐associated fibroblasts represents a promising therapeutic target, as disrupting this pathway through autophagy inhibition or WNT5A neutralization effectively reversed cancer cell malignant behaviors, offering new avenues for overcoming current treatment limitations in colon cancer.

Autophagy regulation in colon cancer involves complex molecular networks including RACK1‐mediated tumor cell survival, microRNAs (miR‐142‐3p, miR‐502‐5p) targeting autophagy genes (Rab1B, ATG5, DHODH), and treatment resistance mechanisms (Xiao et al. 2018; Zheng et al. 2022, Zhai et al. 2013). Autophagy activation contributes to chemotherapy and targeted therapy resistance, as demonstrated by oxaliplatin‐induced autophagy that can be overcome through autophagy inhibitors like chloroquine or Beclin1/ATG5 knockdown (Selvakumaran et al. 2013). Similarly, mTOR inhibitor WYE‐354 combined with autophagy inhibitor 3‐MA enhances anti‐tumor effects, while dual‐target inhibitor DCZ5248 simultaneously blocks Hsp90 and autophagy to induce apoptosis (Chen et al. 2021; Wang et al. 2016). Autophagy‐related gene (ARG) molecular typing has identified distinct colon cancer subtypes with varying immune infiltration patterns and prognosis, with high ATG16L1 expression associated with poor anti‐PD‐L1 response in KRAS‐mutant cases (Taraborrelli et al. 2023; Zhu et al. 2020). Our findings extend this understanding by demonstrating that RCCD1‐mediated autophagy in cancer‐associated fibroblasts creates a protumorigenic microenvironment through WNT5A secretion, and importantly, autophagy inhibition with 3‐MA effectively disrupts this axis, supporting the therapeutic potential of targeting autophagy regulatory networks in colon cancer treatment strategies (Hu et al. 2016).

In this study, we identified RCCD1 as a key autophagy‐related gene in colon cancer, revealing its predominant expression in CAFs and enhanced CAF‐tumor cell interactions. RCCD1, located on chromosome 1q21.3, encodes a protein containing multiple conserved domains that interact with cyclins and kinases to regulate cell cycle progression and proliferation, particularly in rapidly dividing cells under normal physiological conditions (Marcon et al. 2014; Peng et al. 2023). Previous studies have demonstrated significant RCCD1 upregulation in colon cancer tissues at both mRNA and protein levels, with high expression correlating with tumor size, lymph node metastasis, and advanced clinical stage (Han et al. 2024). Mechanistically, RCCD1 promotes colon cancer cell proliferation by regulating cyclins D1 and E while inhibiting apoptosis‐related proteins including Bax and caspase‐3 (Han et al. 2024). Recent evidence suggests RCCD1's involvement in autophagy through coexpression with autophagy‐related gene ATG10 and activation of nonclassical WNT signaling via WNT5A secretion and CaMKII/ERK cascade activation, thereby influencing cell migration, invasion, and epithelial‐mesenchymal transition (Wu et al. 2017; Zhang et al. 2024). Our findings significantly advance this understanding by demonstrating that RCCD1 specifically enhances autophagy in CAFs potentially through AMPK/mTOR/ULK1 signaling, leading to autophagy‐dependent WNT5A secretion that drives cancer cell malignant behaviors, supporting our demonstration that autophagy inhibition effectively disrupts the RCCD1‐autophagy‐WNT5A axis.

WNT5A, a critical ligand in noncanonical WNT signaling, has emerged as a key mediator of cancer progression and epithelial‐mesenchymal transition across various malignancies. In colorectal cancer, WNT5A exhibits context‐dependent roles, functioning both as a tumor suppressor in early stages and as a promoter of metastasis in advanced disease (Pashirzad et al. 2021). Recent studies have specifically highlighted the importance of WNT5A secretion from cancer‐associated fibroblasts in driving colorectal cancer progression. Hirashima et al. demonstrated that WNT5A expression in CAFs significantly promotes colorectal cancer cell migration and invasion through activation of noncanonical WNT pathways (Hirashima et al. 2021). Furthermore, Harada et al. revealed that hypoxic conditions induce WNT5A secretion from fibroblasts, creating a protumorigenic microenvironment that enhances colon cancer progression through paracrine signaling mechanisms (Harada et al. 2025). The molecular mechanism underlying WNT5A‐mediated EMT involves activation of downstream effectors including CaMKII and ERK signaling cascades, which promote loss of epithelial characteristics and acquisition of mesenchymal properties (Abedini et al. 2020). Our findings significantly advance this understanding by demonstrating that RCCD1‐mediated autophagy in CAFs serves as a novel upstream regulator of WNT5A secretion, establishing a previously uncharacterized regulatory axis where autophagy controls the paracrine release of this critical EMT inducer. This mechanistic insight reveals how the tumor microenvironment drives cancer progression, providing therapeutic rationale for targeting the RCCD1‐autophagy‐WNT5A pathway.

While our study provides novel insights into the RCCD1‐autophagy‐WNT5A axis in colon cancer progression, several limitations warrant consideration. First, cancer‐associated fibroblasts exhibit substantial heterogeneity within the tumor microenvironment, with recent studies identifying multiple distinct CAF subtypes characterized by different marker expression profiles, functional states, and protumorigenic activities (Cords et al. 2023; Herrera et al. 2013; Yu et al. 2024). In colorectal cancer specifically, CAF subpopulations demonstrate functional heterogeneity with distinct prognostic gene expression signatures and varying capacities to promote angiogenesis, invasion, and metastasis through diverse signaling mechanisms (Herrera et al. 2013; Yu et al. 2024). While our study establishes RCCD1 as a key regulator in CAFs, how RCCD1‐mediated autophagy and WNT5A secretion vary across different CAF subtypes and whether specific CAF subpopulations are primarily responsible for the observed protumorigenic effects remain to be systematically investigated through single‐cell resolution analyses. Second, our mechanistic studies primarily utilized the HCT116 colon cancer cell line for functional validation. Although we incorporated primary CAFs isolated from patient tissues to enhance clinical relevance, validation of the RCCD1‐WNT5A‐mediated effects in additional colon cancer cell lines or patient‐derived organoid models would strengthen the generalizability of our findings across different molecular subtypes of colorectal cancer. Third, our in vitro coculture system, while revealing important paracrine interactions between CAFs and tumor cells, does not fully recapitulate the complex 3D architecture and multicellular interactions present in the actual tumor microenvironment, including contributions from immune cells, endothelial cells, and extracellular matrix components. Fourth, although we demonstrated the therapeutic potential of targeting the RCCD1‐autophagy‐WNT5A axis using 3‐MA and anti‐WNT5A antibodies in vitro, the efficacy, safety, and potential resistance mechanisms of these interventions require rigorous evaluation in preclinical animal models and ultimately in clinical trials. Future studies using patient‐derived xenografts, genetically engineered mouse models, and comprehensive single‐cell multiomics approaches will be essential to address these limitations and translate our findings into effective therapeutic strategies for colon cancer patients.

## 5 Conclusions

**FIGURE 9:**
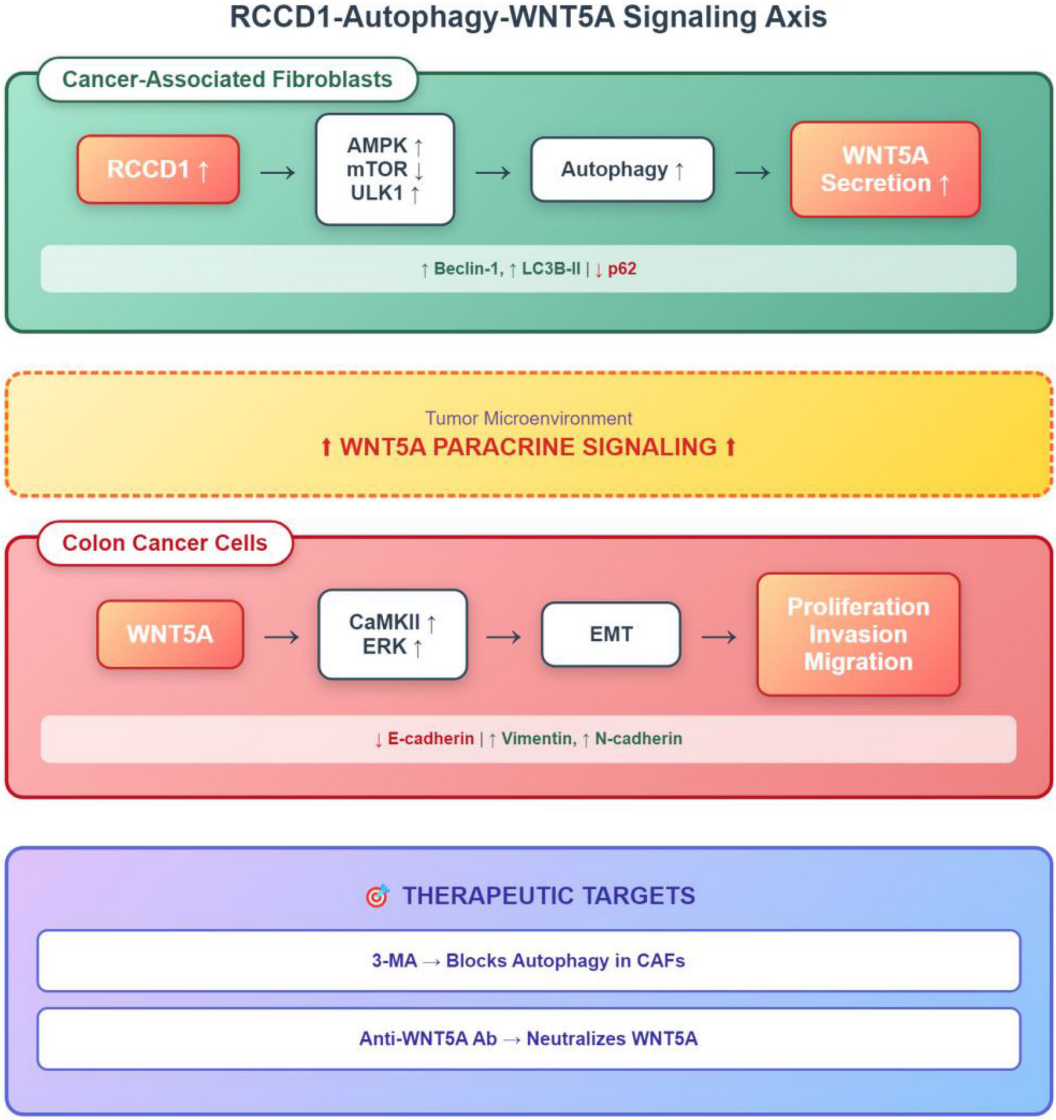
Schematic diagram of the RCCD1‐autophagy‐WNT5A signaling axis mediating CAF‐tumor cell crosstalk in colon cancer progression. In cancer‐associated fibroblasts (CAFs), RCCD1 overexpression activates the AMPK/mTOR/ULK1 signaling cascade, leading to enhanced autophagy characterized by increased Beclin‐1 and LC3B‐II expression and decreased p62 levels. This RCCD1‐mediated autophagy promotes WNT5A secretion from CAFs into the tumor microenvironment. Secreted WNT5A acts as a paracrine factor that binds to receptors on colon cancer cells, activating the noncanonical Wnt/CaMKII/ERK signaling pathway. This activation induces epithelial‐mesenchymal transition (EMT), ultimately enhancing malignant behaviors including proliferation, invasion, and migration. The RCCD1‐autophagy‐WNT5A axis can be therapeutically targeted using 3‐methyladenine (3‐MA) to block autophagy in CAFs or anti‐WNT5A neutralizing antibodies to disrupt paracrine signaling, both of which effectively reverse cancer progression. These data indicate the critical role of the RCCD1‐autophagy‐WNT5A axis in mediating protumorigenic crosstalk between CAFs and tumor cells in the colon cancer microenvironment.

In summary, we used integrated bioinformatics analysis to identify RCCD1 as a key autophagy‐related prognostic gene in colon cancer, with predominant expression in CAFs. Clinical validation confirmed RCCD1 upregulation in CAFs correlates with elevated WNT5A levels. Mechanistically, RCCD1 overexpression enhances autophagic flux potentially through AMPK/mTOR/ULK1 signaling, promoting autophagy‐dependent WNT5A secretion from fibroblasts. Secreted WNT5A activates the noncanonical Wnt/CaMKII/ERK pathway in cancer cells, inducing epithelial‐mesenchymal transition and enhancing malignant behaviors including proliferation, invasion, and migration (Figure 9). Both autophagy inhibitor 3‐MA and anti‐WNT5A neutralizing antibodies effectively reverse these effects. This study establishes the RCCD1‐autophagy‐WNT5A axis as a critical mediator of CAF‐tumor cell crosstalk, providing novel therapeutic targets for disrupting the tumor microenvironment in colon cancer.

## Author Contributions

**Chao Liu**: conceived the study, managed the data and wrote the draft of the manuscript. **Sheng Xu**: collected and analyzed the data. **Yuanyuan Liu**: organize data, pictures, tables. **Yuntian Tang**: conceived the study, revised the manuscript and provided critical feedback on the research methodology. All authors reviewed the manuscript. All authors made substantial contributions to conception and design, acquisition of data, or analysis and interpretation of data; took part in drafting the article or revising it critically for important intellectual content; agreed to submit to the current journal; gave final approval of the version to be published; and agree to be accountable for all aspects of the work.

## Funding

This study was supported by the National Natural Science Foundation of China (81360081).

## Consent

The authors have nothing to report.

## Conflicts of Interest

The authors declare that the research was conducted in the absence of any commercial or financial relationships that could be construed as a potential conflicts of interest.

## Data Availability

The data generated in this study are available upon request to the corresponding author.
